# Significado Clínico do Domínio da Peptidase M20 Contendo 1 em Pacientes com Aterosclerose Carotídea

**DOI:** 10.36660/abc.20210799

**Published:** 2022-04-26

**Authors:** Xincheng Huang, Peiyuan He, Linling Wu

**Affiliations:** 1 Department of the Cardiovascular Medicine Fourth People’s Hospital of Chengdu Chengdu Sichuan China Department of the Cardiovascular Medicine, the Fourth People’s Hospital of Chengdu, Chengdu, Sichuan – China; 2 Health Management Center Sichuan Provincial People’s Hospital Chengdu Sichuan China Health Management Center, Sichuan Provincial People’s Hospital, Chengdu, Sichuan – China; 3 Department of the Second Ward of Acute Psychosis the Fourth People’s Hospital of Chengdu Chengdu Sichuan China Department of the Second Ward of Acute Psychosis, the Fourth People’s Hospital of Chengdu, Chengdu, Sichuan – China

**Keywords:** Doenças das Artérias Carótidas, Lipídeos, Índice de Massa Corporal

## Abstract

**Fundamento:**

A aterosclerose é a principal causa da maioria das doenças cardiovasculares, e novos biomarcadores para essa condição são sempre necessários. O domínio da peptidase M20 contendo 1 (PM20D1) está associado ao metabolismo lipídico e à obesidade. No entanto, nenhum estudo se concentra no papel do PM20D1 na aterosclerose carotídea.

**Objetivo:**

O objetivo deste estudo foi investigar o papel do PM20D1 em pacientes com aterosclerose carotídea.

**Métodos:**

Estudo observacional prospectivo conduzido com um total de 231 pacientes com aterosclerose carotídea que estiveram em nosso departamento entre julho de 2018 e dezembro de 2019. Amostras de sangue e dados médicas foram obtidos de outros 231 indivíduos saudáveis com o mesmo índice de massa corporal (IMC) dos pacientes com aterosclerose carotídea. O PM20D1 sérico foi determinado por ensaio imunossorvente ligado a enzima (ELISA). As características clínicas e demográficas de todos os pacientes foram listadas, incluindo idade, sexo biológico, IMC e histórico médico. Os níveis de proteína C reativa (PCR), fator de necrose tumoral, homocisteína, colesterol total, triglicerídeos, leptina-colesterol de alta densidade e leptina-colesterol de baixa densidade foram registrados. Realizou-se análise estatística no software SPSS, com p<0,05 considerado estatisticamente significante.

**Resultados:**

Os níveis séricos de PM20D1 foram marcadamente mais baixos em pacientes com aterosclerose carotídea comparados aos controles saudáveis, sendo significativamente mais baixos em pacientes com aterosclerose carotídea grave e pacientes com aterosclerose carotídea/acidente vascular cerebral. Pacientes com placas instáveis apresentaram PM20D1 marcadamente menor quando comparados a pacientes com placas estáveis. Nenhuma diferença significativa foi encontrada entre pacientes com aterosclerose carotídea com diferentes IMC. Pacientes com níveis mais elevados de PM20D1 apresentaram expressão significativamente menor de PCR, fator de necrose tumoral, homocisteína, triglicerídeos, colesterol total e colesterol de baixa densidade. PM20D1 correlacionou-se negativamente com PCR, fator de necrose tumoral, homocisteína, colesterol total e leptina de baixa densidade em pacientes com aterosclerose carotídea, podendo ser usado como biomarcador para pacientes com aterosclerose carotídea grave ou com aterosclerose carotídea e acidente vascular cerebral. Sexo biológico, fator de necrose tumoral, homocisteína e PM20D1 foram considerados fatores de risco para aterosclerose carotídea.

**Conclusão:**

O PM20D1 estava diminuído em pacientes com aterosclerose carotídea e foi associado com gravidade, estabilidade da placa, níveis de PCR, fator de necrose tumoral, homocisteína, triglicerídeos, colesterol total e colesterol de baixa densidade em pacientes com aterosclerose carotídea.

## Introdução

Como principal causa da maioria das doenças cardiovasculares, a aterosclerose pode ocorrer precocemente e permanecer assintomática por longos períodos antes de se manifestar.^1,2^ Dentre seus diversos tipos, acredita-se que a aterosclerose carotídea (AC) seja um preditor de acidente vascular cerebral (AVC) isquêmico.^3^ Relata-se que a placa carotídea é um fator de risco para acidente vascular cerebral isquêmico, que também está associado ao cádmio, enquanto o aumento do espessamento médio-intimal (EMI) da carótida e a presença de placa carotídea estão associados ao aumento do risco de acidente vascular cerebral isquêmico em indivíduos com fibrilação atrial.^4,5^ Muitos fatores de risco estão associados à AC, incluindo disfunção do metabolismo lipídico,^6^ hipertensão,^7^ diabetes,^8^ idade,^9^ tabagismo^10^ etc. No entanto, ainda é necessário um entendimento mais profundo sobre a manifestação da AC e seus resultados clínicos.

O domínio da peptidase M20 contendo 1 (PM20D1) é uma enzima secretada, recentemente identificada, que é rica em proteína de desacoplamento 1 (UCP1+), em relação a adipócitos UCP1.^11^ Um estudo recente mostrou que o PM20D1 está ligado ao metabolismo lipídico e pode estar associado à obesidade,^12^ ambos fatores de risco para AC. Verificou-se também que o PM20D1 poderia regular os desacopladores de aminoácidos lipidados das mitocôndrias e aumentar o gasto energético de PM20D1.^13^ No entanto, até o momento, nenhum estudo focou no papel do PM20D1 na AC.

Este é um estudo observacional prospectivo cujo intuito foi investigar o significado clínico do PM20D1 em pacientes com AC. Esta pesquisa pode fornecer evidências clínicas sobre o papel do PM20D1 na AC.

## Materiais e métodos

### Pacientes

Este estudo observacional prospectivo recrutou 231 pacientes com AC assistidos em nosso departamento entre julho de 2018 e dezembro de 2019. Os critérios de inclusão foram: 1) todos os pacientes diagnosticados com AC de acordo com achados da ultrassonografia com Doppler colorido dos vasos sanguíneos do pescoço e angiotomografia computadorizada intracraniana (ATC) do arco aórtico; 2) Pacientes com AC e AVC foram admitidos em até 72 horas após a manifestação, e o diagnóstico de AVC foi confirmado por ressonância magnética (RM) e tomografia computadorizada (TC); 3) os pacientes concordaram em participar da pesquisa observacional. Os critérios de exclusão foram: 1) pacientes com aneurisma cerebral, malformação arteriovenosa, dissecção, arterite ou doença de Moyamoya; 2) pacientes com embolia cerebral cardiogênica; 3) pacientes com outras doenças graves do sistema, incluindo câncer e disfunção cardíaca, renal ou hepática. A gravidade da AC foi definida da seguinte forma: 1) grupo AC leve/moderado, que apresentou espessamento médio-intimal (EMI)>1,0 mm, ou placa com estenose arterial <70% e sem AVC; 2) grupo AC grave, que apresentou uma ou mais placas com estenose arterial ≥70% e sem AVC; 3) grupo AC combinado com AVC, apresentando AC combinado com AVC isquêmico aterosclerótico de grandes artérias e estenose vascular cerebral >50%. As placas foram divididas em estáveis e instáveis, conforme amplamente aceito na prática clínica: 1) EMI≥1,2 mm foi considerado uma placa; 2) as placas estáveis possuem gradiente-eco forte uniforme ou gradiente-eco médio; 3) as placas instáveis são moles ou ulcerativas com gradiente-eco misto ou baixo.^14^ O EMI

foi medido por meio de um instrumento de diagnóstico por ultrassom LOGIQ C9 Color Doppler (General Electric, Estados Unidos da América) com frequência de sonda de 7~14 MHz. Foram medidos EMI da artéria carótida comum bilateral, proximal, distal e a 1 cm da bifurcação da artéria carótida comum. Todas as medidas foram realizadas pelo menos três vezes e o valor médio foi considerado o valor final do EMI.

Além disso, os pacientes foram divididos em diferentes grupos de índice de massa corporal (IMC): grupo normal, com IMC<24 kg/m^2^; grupo com sobrepeso, com 28 kg/m^2^>IMC≥24 kg/m^2^; e grupo obesidade, com IMC>28 kg/m^2^.^15^ Também foram obtidas amostras de sangue e características médicas de 231 indivíduos saudáveis que compareceram ao exame físico de rotina, tendo a mesma distribuição de IMC dos pacientes com AC. Todos os pacientes assinaram o termo de consentimento livre e informado. Este estudo foi aprovado pelo comitê de ética do Fourth People’s Hospital of Chengdu (CDH-2018-057).

### Medição do PM20D1

As amostras de sangue de todos os casos foram coletadas em até 24 horas após a admissão ou chegada ao ambulatório. Resumidamente, 5 ml de sangue foram coletados em tubos sem qualquer anticoagulante. Após uma hora, a centrifugação foi realizada a 2.000 g por 15 minutos, em temperatura ambiente, e foram obtidas as amostras de soro. Os níveis séricos de PM20D1 foram medidos por ensaio imunoenzimático (ELISA), usando um kit PM20D1 (MYBioSource, cat. nº MBS280518), em total acordo com as instruções do fabricante.

### Coleta e medição de dados

As características clínicas e demográficas de todos os pacientes foram coletadas, incluindo idade, sexo biológico, IMC e histórico médico. Foi realizado hemograma total de encaminhamento com um analisador bioquímico automático (Hitachi 7600, Hitachi Corporation, Japão), bem como dosados os níveis de proteína C reativa (PCR), fator de necrose tumoral (TNF-α), homocisteína (Hcy), colesterol total (CT), triglicerídeos (TG), colesterol de leptina de alta densidade (HDL-ch) e colesterol de leptina de baixa densidade (LDL-ch).

### Análise estatística

Os dados com distribuição normal foram expressos por média ± desvio padrão (DP), e os dados com distribuição não normal foram expressos como mediana (intervalo interquartil). As variáveis categóricas foram apresentadas como número (taxas). A distribuição dos dados foi analisada pelo método de Kolmogorov-Smirnov. Para dados com distribuição normal, foi realizada a comparação entre dois grupos pelo teste t não pareado e por análise de variância unidirecional (ANOVA), seguidos pelo teste post hoc de Tukey, usado para comparação entre três ou mais grupos. Para dados com distribuição não normal, utilizou-se o teste de Mann-Whitney para comparação entre dois grupos e o teste de Kruskal-Wallis para comparação entre três ou mais grupos, seguidos do teste post hoc de Dunn. As taxas foram analisadas pelo teste qui-quadrado. A correlação entre PM20D1, Hcy, metabolismo lipídico e fatores inflamatórios foi analisada por meio da correlação de Pearson. Uma curva receiver operating characteristic (ROC) foi usada para análise de PM20D1 em pacientes com AC. A regressão logística foi utilizada para análise de risco de AC. O valor de p<0,05 foi considerado estatisticamente significativo. Todo o cálculo foi realizado no programa SPSS 25.0 (SPSS Inc., Chicago, Estados Unidos da América) ou no GraphPad 6.0 (GraphPad Software, San Diego, Califórnia, Estados Unidos da América).

## Resultados

### Características clínicas basais de todos os pacientes

Esta pesquisa incluiu 231 pacientes com AC no total, sendo 152 casos de AC leve/moderada, 50 casos de AC grave e 29 casos de AC com AVC. A razão da soma dos pacientes com sobrepeso e obesidade foi significativamente maior nos pacientes com AC grave quando comparada à dos pacientes leves/moderados (Tabela 1). A proporção de placa instável foi significativamente maior em pacientes com AC grave e AC/AVC. Os níveis de PCR, TNF-α e Hcy foram notadamente maiores em pacientes com AC grave e pacientes com AC/AVC em comparação aos pacientes leves/moderados. Apenas TG, TC e LDL-ch estavam mais altos em pacientes com AC grave e pacientes com AC/AVC em relação aos casos leves/moderados. Além disso, os controles saudáveis apresentaram níveis significativamente mais baixos de PCR, TNF-α e Hcy, bem como TG, CT e LDL-ch em relação a todos os pacientes com AC. Nenhuma outra diferença significativa foi encontrada.

**Tabela 1:** – Características clínicas basais de todos os pacientes

Variáveis	Todas as ACs, n=231	AC leve/moderada, n=152	AC grave, n=50	AC/AVC, n=29	Saudáveis, n=231	P1*	P2^#^
**Idade, anos**	57,85±9,04	58,17±9,35	58,32±8,90	55,37±7,37	58,36±8,97	0,543	0,289
**Sexo, feminino (%)**	105 (45,45)	64 (42,11)	25 (50,00)	16 (55,17)	108 (46,75)	0,854	0,177
**IMC, n (%)**						0,968	0,176
<24 kg/m^2^	98 (42,42)	65 (42,76)	15 (30,00)	8 (27,59)	102 (44,16)		
28 kg/m^2^>IMC≥24 kg/m^2^	76 (32,90)	48 (31,58)	20 (40,00)	11 (37,93)	73 (31,60)		
>28 kg/m^2^	57 (24,68)	39 (25,66)	15 (30,00)	10 (34,48)	56 (24,24)		
**Complicações, n (%)**						-	0,993
Diabetes	62 (26,84)	39 (25,66)	15 (30,00)	8 (27,59)	-		0,790
Hipertensão	114 (49,35)	75 (49,34)	24 (48,00)	15 (51,72)	-		0,868
Histórico de doença cardíaca coronária	41 (17,75)	27 (17,76)	9 (18,00)	5 (17,24)	-		0,990
Histórico de AVC	12 (5,19)	7 (4,61)	3 (6,00)	2 (6,90)	-		0,804
Tabagismo	129 (55,84)	87 (57,24)	24 (48,00)	18 (62,07)	97 (41,99)	0,090	0,126
**Placa, n (%)**					-	-	<0,001
Estável	103 (44,59)	81 (53,29)	17 (34,00)	5 (17,24)	-		
Instável	128 (55,41)	71 (46,71)	33 (66,00)	24 (82,76)	-		
**PCR, mg/L**	6,91 (0,52~34,87)	5,13 (0,52~9,99)	17,01 (2,92~34,87)	13,40 (3,01~33,64)	4,23 (0,57~10,01)	<0,001	<0,001
**TNF-α, pg/ml**	24,18±12,99	17,45±4,40	37,01±14,70	37,34±13,59	14,81±2,81	<0,001	<0,001
**Hcy, μmol/L**	12,28±3,22	10,78±2,34	15,08±2,72	15,33±2,61	7,46±1,45	<0,001	<0,001
**TC, mmol/L**	4,26±0,78	4,00±0,66	4,80±0,72	4,66±0,84	3,96±0,68	<0,001	<0,001
**TG, mmol/L**	1,48±0,51	1,37±0,54	1,65±0,40	1,77±0,36	1,28±0,45	<0,001	<0,001
**LDL-ch, mmol/L**	3,01±0,55	2,87±0,44	3,25±0,61	3,33±0,66	2,74±0,41	<0,001	<0,001
**HDL-ch, mmol/L**	1,15±0,08	1,15±0,07	1,16±0,08	1,15±0,09	1,16±0,08	0,473	0,735

*Comparação P1 entre todas as ateroscleroses carotídeas (ACs) e o controle saudável. #P2 comparação entre pacientes leves/moderados, graves e AC/AVC. A análise de variância unidirecional seguida pelo teste post hoc de Tukey foi usada para dados com distribuição normal. Para dados com distribuição não normal, o teste de Kruskal-Wallis foi usado para comparação entre pacientes leves/moderados, graves e AC/AVC, seguido do teste post hoc de Dunn. As taxas foram analisadas por meio do teste qui-quadrado. AVC: acidente vascular cerebral; IMC: índice de massa corporal; PCR: proteína C reativa; TNF-α: fator de necrose tumoral; Hcy: homocisteína; CT: colesterol total; TG: triglicérides; LDL-ch: colesterol de leptina de baixa densidade; HDL-ch: colesterol de leptina de alta densidade.

### PM20D1 associado à gravidade e estabilidade da placa em pacientes com AC

**Figura 1:**
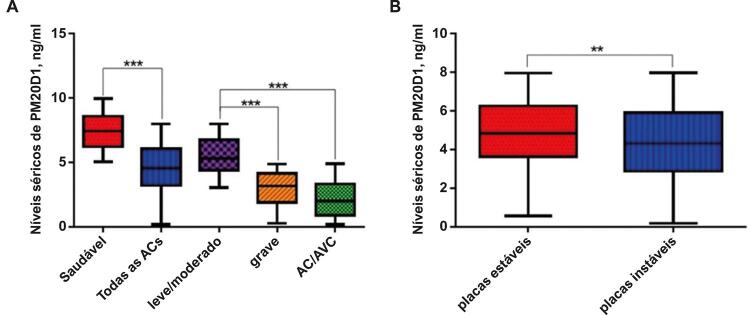
– Níveis séricos de PM20D1 em pacientes com aterosclerose carotídea (AC) com gravidades e estabilidade da placa diferentes. ***p<0,001, **p<0,01. AVC: acidente vascular cerebral. AVC: acidente vascular cerebral.

Em seguida, foi determinada a expressão sérica de PM20D1 em pacientes com AC. Verificaram-se níveis séricos de PM20D1 marcadamente mais baixos em pacientes com AC quando comparados ao controle saudável (Figura 1). Pacientes com AC grave e com AC/AVC apresentaram níveis significativamente mais baixos de PM20D1 em comparação aos pacientes leves/moderados. No entanto, nenhuma diferença significativa foi encontrada entre pacientes graves e AC/AVC. Pacientes com placas instáveis apresentaram expressão marcadamente mais baixa de PM20D1 do que pacientes com placas estáveis.

### PM20D1 não associado a pacientes com aterosclerose carotídea e diferentes índices de massa corporal

**Figura 2:**
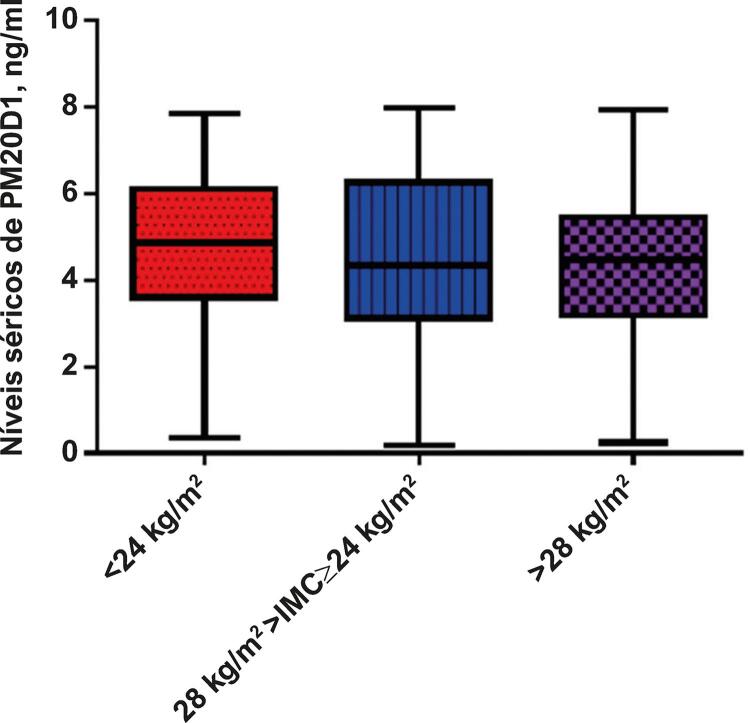
– Níveis séricos de PM20D1 em pacientes com aterosclerose carotídea e diferentes índices de massa corporal (IMC).

Para investigar mais detalhadamente o papel do PM20D1 em pacientes com AC, também avaliamos os níveis de PM20D1 em pacientes com AC e diferentes distribuições de IMC. Não foi observada diferença significativa (Figura 2).

### Correlação entre PM20D1, metabolismo lipídico e fatores inflamatórios

Em seguida, os pacientes foram divididos em grupo de alta expressão de PM20D1 e grupo de baixa expressão, de acordo com o nível médio de PM20D1 (4,53 ng/ml). Pacientes com níveis mais elevados de PM20D1 apresentaram expressão significativamente menor de PCR, TNF-α, Hcy, TG, CT e LDL-ch (Tabela 2). Foi feita a correlação de Pearson e mostrou que o PM20D1 se correlacionou negativamente com PCR, TNF-α, Hcy, TC e LDL-ch em pacientes com AC (Tabela 3).

**Tabela 2:** – Expressão do metabolismo lipídico e fatores inflamatórios em pacientes com diferentes níveis de PM20D1

Variáveis	PM20D1 alto, n=117	PM20D1 baixo, n=114	p*
PCR, mg/L	5,27 (0,52~33,64)	15,16 (0,68~34,87)	<0,001
TNF-α, pg/ml	18,78±7,32	29,72±15,08	<0,001
Hcy, μmol/L	11,26±2,66	13,34±3,41	<0,001
CT, mmol/L	4,03±0,70	4,49±0,80	<0,001
TG, mmol/L	1,41±0,54	1,56±0,47	0,031
LDL-ch, mmol/L	2,90±0,48	3,12±0,59	0,003
HDL-ch, mmol/L	1,14±0,08	1,16±0,08	0,269

*O teste t de Student foi usado para comparar pacientes leves/moderados, graves e AC/AVC para dados normalmente distribuídos. Para dados com distribuição não normal, utilizou-se o teste de Mann-Whitney. As taxas foram analisadas pelo teste qui-quadrado. PCR: proteína C reativa; TNF-α: fator de necrose tumoral; Hcy: homocisteína; CT: colesterol total; TG: triglicérides; LDL-ch: colesterol de leptina de baixa densidade; HDL-ch: colesterol de leptina de alta densidade.

**Tabela 3:** – Correlação entre PM20D1, homocisteína (Hcy), metabolismo lipídico e fatores inflamatórios

Variáveis	Correlação de Pearson	p
PCR, mg/L	-0,514	<0,001
TNF-α, pg/ml	-0,585	<0,001
Hcy, μmol/L	-0,598	<0,001
TC, mmol/L	-0,254	<0,001
TG, mmol/L	-0,059	0,198
LDL-ch, mmol/L	-0,071	0,126
HDL-ch, mmol/L	0,022	0,622

*PCR: proteína C reativa; TNF-α: fator de necrose tumoral; Hcy: homocisteína; CT: colesterol total; TG: triglicérides; LDL-ch: colesterol de leptina de baixa densidade; HDL-ch: colesterol de leptina de alta densidade.*

### Valor diagnóstico de PM20D1 na aterosclerose carotídea

**Figura 3:**
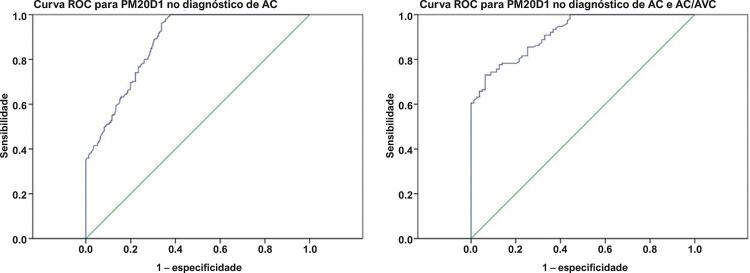
– Curva receiver operating characteristic (ROC) para valor diagnóstico de PM20D1 de aterosclerose carotídea (AC), AC grave ou AC/AVC. AVc: acidente vascular cerebral.

Por fim, uma curva ROC foi desenhada para observar o valor diagnóstico de PM20D1 para AC, bem como AC grave ou AC/AVC. Verificou-se que o PM20D1 poderia ser um potencial biomarcador diagnóstico de AC com valor de corte de 5,94 ng/ml, área sob a curva ROC (AUC) 0,876, IC95% (0,845~0,906), sensibilidade de 80,1% e especificidade de 73,6% (Figura 3). O PM20D1 também pode ser usado como biomarcador para pacientes com AC grave ou AC/AVC, com valor de corte de 3,99 ng/ml, AUC 0,917, IC95% (0,883~0,951), sensibilidade de 81,6%, especificidade de 77,2%. A regressão logística binária adicional, que incluiu todos os fatores de idade, IMC, PCR, TNF-α, Hcy, TC, TG, LDL-ch, HDL-ch, PM20D1, bem como sexo biológico, diabetes, hipertensão, histórico de doença cardíaca coronária, histórico de AVC e tabagismo, estabeleceu sexo, TNF-α, Hcy e PM20D1 como fatores de risco para AC (Tabela 4).

**Tabela 4:** – Regressão logística binária para fatores de risco de aterosclerose carotídea

Variáveis	Wald	Odds ratio	IC95%	p
Idade	0,011	1,003	0,935~1,078	0,914
Sexo	4,210	0,208	1,073~21,401	0,040
IMC	0,524	1,057	0,909~1,231	0,469
Diabetes	<0,001	<0,001	0,000	0,995
Hipertensão	<0,001	<0,001	0,000	0,993
Histórico de doença cardíaca coronária	<0,001	<0,001	0,000	0,995
Histórico de AVC	<0,001	0,001	0,000	0,999
Tabagismo	<0,001	0,844	0,232~3,075	0,798
PCR	0,004	1,008	0,777~1,310	0,947
TNF-α	11,820	0,664	0,526~0,839	0,001
Hcy	22,852	0,155	0,073~0,334	<0,001
CT	0,462	0,697	0,248~1,967	0,496
TG	1,403	0,411	0,095~1,787	0,236
LDL-ch	1,559	0,340	0,063~1,847	0,212
HDL-ch	1,216	0,008	1,797E-6~40,532	0,270
PM20D1	18,152	8,485	3,173~22,693	<0,001

*IMC: índice de massa corporal; AVC: acidente vascular cerebral; PCR: proteína C reativa; TNF-α: fator de necrose tumoral; Hcy: homocisteína; CT: colesterol total; TG: triglicérides; LDL-ch: colesterol de leptina de baixa densidade; HDL-ch: colesterol de leptina de alta densidade.*

## Discussão

O diagnóstico precoce e predição da AC podem melhorar muito a eficácia e o prognóstico do tratamento. Assim, biomarcadores para diagnóstico e prognóstico da AC são de grande importância. Neste estudo, demonstramos que PM20D1 estava mais baixo em pacientes com AC e se associou com a gravidade e condição de placa dos pacientes com AC. Além disso, o PM20D1 correlacionou-se negativamente com PCR, TNF-α, Hcy, TC e LDL-ch nesses pacientes.

O metabolismo lipídico e a obesidade estão associados ao desenvolvimento da AC. Verificou-se que os níveis de TG, TC e LDL-ch estavam significativamente maiores em pacientes com placa na artéria carótida.^16^ Outra pesquisa constatou que o tratamento com atorvastatina ou ezetimiba diminuiu os níveis de TC, TG e LDL-ch em pacientes com AC.^17^ Em estudo recente, Pan et al. mostraram que, em residentes rurais de baixa renda na China, LDL-ch e TC foram fatores de risco para aterosclerose em estágio inicial e foram ligados ao risco aumentado de placa carotídea em 24% e 62% para cada aumento de 1 mmol/L em TC e LDL-ch.^18^ Em nossa pesquisa, também encontramos que TG, TC e LDL-ch foram expressos em níveis altos em pacientes com AC, principalmente em casos graves, corroborando as pesquisas supracitadas. Também observamos que fatores inflamatórios como PCR, TNF-α e expressão de Hcy estavam elevados em pacientes com AC, o que também foi demonstrado em diversas pesquisas.^19-22^

O PM20D1 é um fator associado tanto ao metabolismo lipídico quanto à obesidade. Verificou-se que camundongos com aumento de PM20D1 circulante apresentaram aumento da frequência respiratória e aumento sérico dos aminoácidos N-acil, o que melhorou a homeostase da glicose e aumentou o consumo de energia e, portanto, pode regular a obesidade.^13^ Benson et al. demonstraram que a diminuição do PM20D1 estava associada à relação genética entre obesidade e doenças neurodegenerativas em humanos.^23^ Li et al. descobriram que a inibição do microRNA miR-324-5p aumentou o consumo de oxigênio das células primárias dos tecidos adiposos branco e marrom, aumentou o consumo de gordura e, assim, reduziu o peso dos camundongos, aumentando os níveis de PM20D1.^24^ Long et al. observaram que, em camundongos com deficiência de PM20D1, a atividade da hidrolase/sintetase do N-acetil aspartato estava diminuída no soro e nos tecidos, bem como uma variedade de fenótipos metabólicos e de dor, incluindo resistência à insulina, mudanças de temperatura fria e comportamento antinociceptivo, que estão associados à obesidade, diabetes e outras doenças.^12^ Assim, podemos especular que níveis mais altos de PM20D1 podem ser benéficos para a obesidade e redução de lipídios. Embora não haja nenhum estudo que demonstre o papel de PM20D1 na AC, a regulação diminuída de PM20D1 em pacientes com AC em nosso estudo pode se dever, em parte, à disfunção do metabolismo lipídico e obesidade de pacientes com AC. Como os pacientes apresentaram níveis mais baixos de PM20D1 do que os controles saudáveis com mesma distribuição de IMC, e os pacientes com AC e IMC diferente não apresentaram diferença significativa de PM20D1; ou seja, o IMC pode não estar associado ao nível de PM20D1 em pacientes com AC. A correlação negativa entre PM20D1 e Hcy, TC e LDL-ch pode ser uma das razões para a expressão anormal de PM20D1 em pacientes com AC. No entanto, para fundamentar todas essas especulações, são necessários mais estudos para obter mais evidências.

O presente estudo teve, no entanto, algumas limitações. Primeiro, trata-se de um estudo observacional de um único centro, com apenas 231 casos. Assim como o mecanismo molecular do PM20D1 exercendo influência no desenvolvimento da AC ainda não está claro.

## Conclusão

Em conclusão, este estudo observacional demonstrou que a diminuição de PM20D1 estava associada à gravidade, condição da placa e expressão de PCR, TNF-α, Hcy, TG, TC e LDL-ch em pacientes com AC. Este estudo pode fornecer um novo alvo de pesquisa para PM20D1 em relação à AC.

## References

[1] Song P, Fang Z, Wang H, Cai Y, Rahimi K, Zhu Y (2020). Global and Regional Prevalence, Burden, and Risk Factors for Carotid Atherosclerosis: A Systematic Review, Meta-analysis, and Modelling Study. Lancet Glob Health.

[2] Tzoulaki I, Castagné R, Boulangé CL, Karaman I, Chekmeneva E, Evangelou E (2019). Serum Metabolic Signatures of Coronary and Carotid Atherosclerosis and Subsequent Cardiovascular Disease. Eur Heart J.

[3] Parish S, Arnold M, Clarke R, Du H, Wan E, Kurmi O (2019). Assessment of the Role of Carotid Atherosclerosis in the Association Between Major Cardiovascular Risk Factors and Ischemic Stroke Subtypes. JAMA Netw Open.

[4] Borné Y, Fagerberg B, Persson M, Östling G, Söderholm M, Hedblad B (2017). Cadmium, Carotid Atherosclerosis, and Incidence of Ischemic Stroke. J Am Heart Assoc.

[5] Bekwelem W, Jensen PN, Norby FL, Soliman EZ, Agarwal SK, Lip GY (2016). Carotid Atherosclerosis and Stroke in Atrial Fibrillation: The Atherosclerosis Risk in Communities Study. Stroke.

[6] Izumi S, Muano T, Mori A, Kika G, Okuwaki S (2006). Common Carotid Artery Stiffness, Cardiovascular Function and Lipid Metabolism After Menopause. Life Sci.

[7] Geraci G, Zammuto M, Gaetani R, Mattina A, D’Ignoto F, Geraci C (2019). Relationship of a Body Shape Index and Body Roundness Index with Carotid Atherosclerosis in Arterial Hypertension. Nutr Metab Cardiovasc Dis.

[8] Carbonell M, Castelblanco E, Valldeperas X, Betriu À, Traveset A, Granado-Casas M (2018). Diabetic Retinopathy is Associated with the Presence and Burden of Subclinical Carotid Atherosclerosis in Type 1 Diabetes. Cardiovasc Diabetol.

[9] Taylor BA, Zaleski AL, Capizzi JA, Ballard KD, Troyanos C, Baggish AL (2014). Influence of Chronic Exercise on Carotid Atherosclerosis in Marathon Runners. BMJ Open.

[10] Cho HM, Kang DR, Kim HC, Oh SM, Kim BK, Suh I (2015). Association Between Fibrinogen and Carotid Atherosclerosis According to Smoking Status in a Korean Male Population. Yonsei Med J.

[11] Sanchez-Mut JV, Heyn H, Silva BA, Dixsaut L, Garcia-Esparcia P, Vidal E (2018). PM20D1 is a Quantitative Trait Locus Associated with Alzheimer’s Disease. Nat Med.

[12] Long JZ, Roche AM, Berdan CA, Louie SM, Roberts AJ, Svensson KJ (2018). Ablation of PM20D1 Reveals N-acyl Amino Acid Control of Metabolism and Nociception. Proc Natl Acad Sci U S A.

[13] Long JZ, Svensson KJ, Bateman LA, Lin H, Kamenecka T, Lokurkar IA (2016). The Secreted Enzyme PM20D1 Regulates Lipidated Amino Acid Uncouplers of Mitochondria. Cell.

[14] Funakoshi Y, Imamura H, Tani S, Adachi H, Fukumitsu R, Sunohara T (2020). Safety and Efficacy of an Open-cell Stent and Double-balloon Protection for Unstable Plaques: Analysis of 184 Consecutive Carotid Artery Stentings. J Neurointerv Surg.

[15] Wang Y, Wang K, Han T, Zhang P, Chen X, Wu W (2020). Exposure to Multiple Metals and Prevalence for Preeclampsia in Taiyuan, China. Environ Int.

[16] Feng S, Zhu Y, Yan C, Wang Y, Zhang Z (2015). Retinol Binding Protein 4 Correlates with and is an Early Predictor of Carotid Atherosclerosis in Type 2 Diabetes Mellitus Patients. J Biomed Res.

[17] Wang J, Ai XB, Wang F, Zou YW, Li L, Yi XL (2017). Efficacy of Ezetimibe Combined with Atorvastatin in the Treatment of Carotid Artery Plaque in Patients with Type 2 Diabetes Mellitus Complicated with Coronary Heart Disease. Int Angiol.

[18] Pan J, Liu J, Wang H, Li W, Du X, Lin Q (2020). Association of Carotid Atherosclerosis With Lipid Components in Asymptomatic Low-Income Chinese: A Population-Based Cross-Sectional Study. Front Neurol.

[19] Wu MM, Chiou HY, Hsueh YM, Hong CT, Su CL, Chang SF (2006). Effect of Plasma Homocysteine Level and Urinary Monomethylarsonic Acid on the Risk of Arsenic-associated Carotid Atherosclerosis. Toxicol Appl Pharmacol.

[20] Lorenz MW, Karbstein P, Markus HS, Sitzer M (2007). High-sensitivity C-reactive Protein is not Associated with Carotid Intima-media Progression: The Carotid Atherosclerosis Progression Study. Stroke.

[21] Fawzy RM, Hammad GA, Egila SE, Elkasas AN, Fouad NA (2020). Association of Tumor Necrosis factor-α (TNF-α)− 308A/G (rs1800629) Gene Polymorphism with Carotid Artery Atherosclerosis in Rheumatoid Arthritis Patients. Egypt Rheumatol.

[22] Zardi EM, Pipita ME, Giorgi C, Lichinchi D, Zardi DM, Afeltra A (2018). Differences in Carotid Atherosclerosis Between Patients with Ankylosing Spondylitis Treated with Tumor Necrosis Factor-α Antagonists and Healthy Matched Controls. Medicine.

[23] Benson KK, Hu W, Weller AH, Bennett AH, Chen ER, Khetarpal SA (2019). Natural Human Genetic Variation Determines Basal and Inducible Expression of PM20D1, an Obesity-associated Gene. Proc Natl Acad Sci U S A.

[24] Li D, Liu Y, Gao W, Han J, Yuan R, Zhang M (2019). Inhibition of miR-324-5p Increases PM20D1-mediated White and Brown Adipose Loss and Reduces Body Weight in Juvenile Mice. Eur J Pharmacol.

