# Supplementary material for: Significado Clínico do Domínio da Peptidase M20 Contendo 1 em Pacientes com Aterosclerose Carotídea
Source: Arq Bras Cardiol. 2022 Apr 26;119(3):372–9. [Article in Portuguese] doi: 10.36660/abc.20210799 (PMC9438548; doi:10.36660/abc.20210799)
Supplement: Translation

# Translation: Clinical Significance of Peptidase M20 Domain Containing 1 Ii Patients with Carotid Atherosclerosis

**Authors:** Xincheng Huang, Peiyuan He, Linling Wu

**Affiliations:** 1 China Department of the Cardiovascular Medicine, the Fourth People’s Hospital of Chengdu, Chengdu, Sichuan – China; 2 China Health Management Center, Sichuan Provincial People’s Hospital, Chengdu, Sichuan – China; 3 China Department of the Second Ward of Acute Psychosis, the Fourth People’s Hospital of Chengdu, Chengdu, Sichuan – China

**Keywords:** Carotid Artery Diseases, Lipids, Body Mass Index

## Abstract

**Background:**

Atherosclerosis is the main cause for most cardiovascular diseases, and new biomarkers for this condition are always needed. Peptidase M20 domain containing 1 (PM20D1) is associated with both lipid metabolism and obesity. However, no study focuses on the role of PM20D1 in carotid atherosclerosis.

**Objective:**

The present study aimed to investigate the role of PM20D1 in carotid atherosclerosis patients.

**Methods:**

The present prospective observational study contained a total of 231 carotid atherosclerosis patients, who went to our department between July 2018 and December 2019. Blood samples and medical characteristics were also obtained from 231 healthy individuals with the same body mass index distribution of carotid atherosclerosis patients. Serum PM20D1 was determined using enzyme-linked immunosorbent assay. Clinical and demographic characteristics of all patients were collected, including age, sex, body mass index and medical history. Levels of C-reactive protein, tumor necrosis factor, homocysteine, as well as total cholesterol, triglyceride, high-density leptin cholesterol and low-density leptin cholesterol were recorded. Statistical analysis was conducted using the SPSS software, with p<0.05 as statistically different.

**Results:**

Serum PM20D1 levels were markedly lower in carotid atherosclerosis patients when compared to the healthy control, which were significantly lower in severe carotid atherosclerosis patients and carotid atherosclerosis/stroke patients. Patients with unstable plaques showed markedly lower PM20D1 when compared to patients with stable plaques. No significant difference was found among carotid atherosclerosis patients with different body mass index. Patients with higher PM20D1 levels showed significantly lower expression of C-reactive protein, tumor necrosis factor, homocysteine, triglyceride, total cholesterol and low-density leptin cholesterol. PM20D1 was negatively correlated with C-reactive protein, tumor necrosis factor, homocysteine, total cholesterol and low-density leptin cholesterol in carotid atherosclerosis patients, and could be used as a biomarker for severe carotid atherosclerosis patients or carotid atherosclerosis patients with stroke. Sex, tumor necrosis factor, homocysteine and PM20D1 were risk factors for carotid atherosclerosis.

**Conclusion:**

PM20D1 was decreased in carotid atherosclerosis patients and was associated with severity, plaque stability, and levels of C-reactive protein, tumor necrosis factor, homocysteine, triglyceride, total cholesterol and low-density leptin cholesterol in carotid atherosclerosis patients.

## Introduction

As the main cause of most cardiovascular diseases, atherosclerosis (AS) can occur early in lifetime and remain asymptomatic for long periods before onset.^1,2^ Among kinds of atherosclerosis, carotid atherosclerosis (CAS) is thought to be a predictor for ischemic stroke.^3^ It is reported that carotid plaque is a risk factor for ischemic stroke, which is also associated with the cadmium, and increased carotid intima-media thickness and presence of carotid plaque are associated with increased risk of ischemic stroke in individuals with atrial fibrillation.^4,5^ Many risk factors are reported to be associated with CAS, including dysfunction of lipid metabolism,^6^ hypertension,^7^ diabetes,^8^ age,^9^ smoking^10^ etc. However, deeper insights are still needed for CAS onset and clinical outcomes.

Peptidase M20 domain containing 1 (PM20D1) is a newly identified secreted enzyme enriched in uncoupling protein 1 (UCP1+), versus UCP1- adipocytes.^11^ A recent study found PM20D1 is associated with lipid metabolism and might be associated with obesity,^12^ which are all risk factors for CAS. It was also found that PM20D1 could regulate lipidated amino acid uncouplers of mitochondria and increased PM20D1 augmented energy expenditure.^13^ However, up to now, no study focuses on the role of PM20D1 in CAS.

The present research performed a prospective observational study in order to investigate the clinical significance of PM20D1 in CAS patients. This research might provide clinical evidence for the role of PM20D1 in CAS.

## Methods and materials

### Patients

The present prospective observational study enrolled a total of 231 CAS patients, who went to our department between July 2018 and December 2019. The inclusion criteria were: 1) all patients were diagnosed as CAS according to color Doppler ultrasound of neck blood vessels and aortic arch intracranial computed tomography angiography (CTA); 2) CAS patients with stroke were admitted within 72 hours after onset, and the diagnosis of stroke was confirmed by magnetic resonance imaging (MRI) and computed tomography (CT) scan; 3) patients agreed to participate in the observational research. The following patients were excluded: 1) patients with cerebral aneurysm, arteriovenous malformation, dissection, arteritis or moyamoya disease; 2) patients with cardiogenic cerebral embolism; 3) patients with other severe system diseases, including cancer and heart, renal or liver dysfunction. The severity of CAS was defined as: 1) mild/moderate CAS group, who showed thickening of the intima-media, with intima-media thickness (IMT) >1.0 mm, or plaque information with arterial stenosis <70% and without stroke; 2) severe CAS group, who showed one or more plaques with arterial stenosis ≥70% and without stroke; 3) CAS combined with stroke group, who showed CAS combined with large artery atherosclerotic ischemic stroke and cerebral vascular stenosis >50%. The plaques were divided into stable and unstable plaques as widely accepted in clinic: 1) IMT≥1.2 mm was considered as plaque; 2) the stable plaques are plaques with uniform strong echo or medium echo; 3) the unstable plaques are soft plaque or ulcerative plaque with mixed echo or low echo.^14^ The measurement of IMT was conducted using a LOGIQ C9 Color Doppler ultrasound diagnostic instrument (General Electric, United States of America) with probe frequency of 7~14 MHz. The IMT of bilateral common carotid artery, proximal, distal and 1 cm from the bifurcation of common carotid artery were measured. All measurement was conducted at least three times and the mean value was considered as the final IMT value.

Additionally, patients were further divided into different body mass index (BMI) groups: the normal group, with BMI<24 kg/m^2^; the overweight group, with 28 kg/m^2^>BMI≥24 kg/m^2^; and the obesity group, with BMI>28 kg/m^2^.^15^ Blood samples and medical characteristics were also obtained from 231 healthy individuals, who came to routine physical examination with the same BMI distribution of CAS patients. All patients signed the informed consent. The present study was approved by the ethical committee of the Fourth People’s Hospital of Chengdu (CDH-2018-057).

### Measurement of PM20D1

The blood samples of all cases were collected within 24 hours of admission or coming to outpatient. Briefly, 5 ml of blood was collected into tubes without any anticoagulant. After 1 hour, centrifugation was conducted at 2,000 g for 15 minutes, in room temperature, and the serum samples were obtained. The serum PM20D1 levels were measured by enzyme-linked immunosorbent assay (ELISA), using a PM20D1 kit (MYBioSource, cat. no. MBS280518), strictly according to the manufacturer’s instruction.

### Data collection and measurement

Clinical and demographic characteristics of all patients were collected, including age, sex, BMI and medical history. Routing whole blood test was performed using an automatic biochemical analyzer (Hitachi 7600, Hitachi Corporation, Japan), and the levels of C-reactive protein (CRP), tumor necrosis factor (TNF-α), homocysteine (Hcy), as well as total cholesterol (TC), triglyceride (TG), high-density leptin cholesterol (HDL-ch) and low-density leptin cholesterol (LDL-ch), were recorded.

### Statistical analysis

Data distributed normally was expressed by mean ± standard deviation (SD), and non-normal distributed data was expressed as median (interquartile range). Categorical variables were shown as number (rates). The distribution of the data was analyzed by Kolmogorov-Smirnov method. For normally distributed data, comparison between two groups was conducted by unpaired t-test and one-way analysis of variance (ANOVA), followed by Tukey’s post hoc test, which was used for comparison among three or more groups. For non-normal distributed data, Mann-Whitney test was used for comparison between two groups, and Kruskal-Wallis test was used for comparison among three or more groups, following with Dunn’s post hoc test. Rates were analyzed using chi-squared test. Correlation among PM20D1, Hcy, lipid metabolism and inflammatory factors was analyzed using the Pearson’s correlation. A receiver operating characteristic (ROC) curve was used for analysis of PM20D1 in CAS patients. Logistic regression was used for analysis of risk of CAS. p<0.05 was considered as statistically different. All calculation was performed using SPSS 25.0 (SPSS Inc., Chicago, United States of America) or GraphPad 6.0 (GraphPad Software, San Diego, California, United States of America).

## Results

### Basic clinical characteristics of all patients

This research included a total of 231 CAS patients, with 152 cases of mild/moderate CAS, 50 cases of severe CAS and 29 cases of CAS with stroke. The ratio of the sum of overweight and obesity patients was significantly higher in severe CAS patients when compared to the mild/moderate patients (Table 1). The ratio of unstable plaque was significantly higher in severe CAS and CAS/stroke patients. The levels of CRP, TNF-α and Hcy were markedly higher in severe CAS patients and CAS/stroke patients when compared to the mild/moderate patients. Only TG, TC and LDL-ch were found to be remarkably higher in severe CAS patients and CAS/stroke patients when compared to the mild/moderate cases. Besides, the healthy control showed

**Table 1:** – Basic clinical characteristics of all patients

Variables	All CAS, n=231	Mild/moderate CAS, n=152	Severe CAS, n=50	CAS/stroke, n=29	Healthy, n=231	P1*	P2^#^
**Age, y**	57.85±9.04	58.17±9.35	58.32±8.90	55.37±7.37	58.36±8.97	0.543	0.289
**Sex, female (%)**	105 (45.45)	64 (42.11)	25 (50.00)	16 (55.17)	108 (46.75)	0.854	0.177
**BMI, n (%)**						0.968	0.176
<24 kg/m^2^	98 (42.42)	65 (42.76)	15 (30.00)	8 (27.59)	102 (44.16)		
28 kg/m2>BMI≥24 kg/m^2^	76 (32.90)	48 (31.58)	20 (40.00)	11 (37.93)	73 (31.60)		
>28 kg/m^2^	57 (24.68)	39 (25.66)	15 (30.00)	10 (34.48)	56 (24.24)		
**Complications, n (%)**						-	0.993
Diabetes	62 (26.84)	39 (25.66)	15 (30.00)	8 (27.59)	-		0.790
Hypertension	114 (49.35)	75 (49.34)	24 (48.00)	15 (51.72)	-		0.868
History of coronary heart disease	41 (17.75)	27 (17.76)	9 (18.00)	5 (17.24)	-		0.990
History of stroke	12 (5.19)	7 (4.61)	3 (6.00)	2 (6.90)	-		0.804
Current smoker	129 (55.84)	87 (57.24)	24 (48.00)	18 (62.07)	97 (41.99)	0.090	0.126
**Plaque, n (%)**					-	-	<0.001
Stable	103 (44.59)	81 (53.29)	17 (34.00)	5 (17.24)	-		
Unstable	128 (55.41)	71 (46.71)	33 (66.00)	24 (82.76)	-		
**CRP, mg/L**	6.91 (0.52~34.87)	5.13 (0.52~9.99)	17.01 (2.92~34.87)	13.40 (3.01~33.64)	4.23 (0.57~10.01)	<0.001	<0.001
**TNF-α, pg/ml**	24.18±12.99	17.45±4.40	37.01±14.70	37.34±13.59	14.81±2.81	<0.001	<0.001
**Hcy, μmol/L**	12.28±3.22	10.78±2.34	15.08±2.72	15.33±2.61	7.46±1.45	<0.001	<0.001
**TC, mmol/L**	4.26±0.78	4.00±0.66	4.80±0.72	4.66±0.84	3.96±0.68	<0.001	<0.001
**TG, mmol/L**	1.48±0.51	1.37±0.54	1.65±0.40	1.77±0.36	1.28±0.45	<0.001	<0.001
**LDL-ch, mmol/L**	3.01±0.55	2.87±0.44	3.25±0.61	3.33±0.66	2.74±0.41	<0.001	<0.001
**HDL-ch, mmol/L**	1.15±0.08	1.15±0.07	1.16±0.08	1.15±0.09	1.16±0.08	0.473	0.735

*P1 comparison between all carotid atherosclerosis (CAS) and the healthy control. #P2 comparison among mild/moderate, severe and CAS/stroke patients. One-way analysis of variance followed by Tukey’s post hoc test was used for normally distributed data. For non-normal distributed data, Kruskal-Wallis test was used for comparison among mild/moderate, severe and CAS/stroke patients, following with Dunn’s post hoc test. Rates were analyzed using chi-squared test. BMI: body mass index; CRP: C-reactive protein; TNF-α: tumor necrosis factor; Hcy: homocysteine; TC: total cholesterol; TG: triglyceride; LDL-ch: low-density leptin cholesterol; HDL-ch: high-density leptin cholesterol.

significantly lower levels of CRP, TNF-α and Hcy, as well as TG, TC and LDL-ch than all CAS patients. No other significant difference was found.

### PM20D1 was associated with severity and plaque stability of CAS patients

Then, the expression of serum PM20D1 in CAS patients was determined. It was found that serum PM20D1 levels were markedly lower in CAS patients when compared to the healthy control (Figure 1). Severe CAS patients and CAS/stroke patients showed significantly lower PM20D1 levels when compared to the mild/moderate patients. However, no significant difference was found between severe and CAS/stroke patients. Additionally, patients with unstable plaques showed markedly lower PM20D1 expression than patients with stable plaques.

**Figure 1:** – Serum levels of PM20D1 in carotid atherosclerosis (CAS) patients with different severity and plaque stability. ***p<0.001, **p<0.01.

PM20D1 was not associated with carotid atherosclerosis patients with different body mass index

To further investigate the role of PM20D1 in CAS patients, we also evaluated the levels of PM20D1 in CAS patients with different BMI distribution. No significant difference was observed among patients with different BMI (Figure 2).

**Figure 2:** – Serum levels of PM20D1 in carotid atherosclerosis (CAS) patients with different body mass index (BMI).

### Correlation between PM20D1, lipid metabolism and inflammatory factors

Then, patients were divided into PM20D1 high expression group and low expression group, according to the mean level of PM20D1 (4.53 ng/ml). It was observed that patients with higher PM20D1 levels showed significantly lower expression of CRP, TNF-α, Hcy, TG, TC and LDL-ch (Table 2). Pearson’s correlation was then conducted and results showed that PM20D1 was negatively correlated with CRP, TNF-α, Hcy, TC and LDL-ch in CAS patients (Table 3).

**Table 2:** – Expression of lipid metabolism and inflammatory factors in patients with different PM20D1

Variables	PM20D1 high, n=117	PM20D1 low, n=114	p*
CRP, mg/L	5.27 (0.52~33.64)	15.16 (0.68~34.87)	<0.001
TNF-α, pg/ml	18.78±7.32	29.72±15.08	<0.001
Hcy, μmol/L	11.26±2.66	13.34±3.41	<0.001
TC, mmol/L	4.03±0.70	4.49±0.80	<0.001
TG, mmol/L	1.41±0.54	1.56±0.47	0.031
LDL-ch, mmol/L	2.90±0.48	3.12±0.59	0.003
HDL-ch, mmol/L	1.14±0.08	1.16±0.08	0.269

*Student’s t-test was used for comparison among mild/moderate, severe and CAS/stroke patients for normally distributed data. For non-normal distributed data, Mann-Whitney test was used for comparison between two groups. Rates were analyzed using chi-squared test. CRP: C-reactive protein; TNF-α: tumor necrosis factor; Hcy: homocysteine; TC: total cholesterol; TG: triglyceride; LDL-ch: low-density leptin cholesterol; HDL-ch: high-density leptin cholesterol.

**Table 3:** – Correlation between PM20D1, homocysteine (Hcy), lipid metabolism and inflammatory factors

Variables	Pearson’s correlation	p value
CRP, mg/L	-0.514	<0.001
TNF-α, pg/ml	-0.585	<0.001
Hcy, μmol/L	-0.598	<0.001
TC, mmol/L	-0.254	<0.001
TG, mmol/L	-0.059	0.198
LDL-ch, mmol/L	-0.071	0.126
HDL-ch, mmol/L	0.022	0.622

*CRP: C-reactive protein; TNF-α: tumor necrosis factor; Hcy: homocysteine; TC: total cholesterol; TG: triglyceride; LDL-ch: low-density leptin cholesterol; HDL-ch: high-density leptin cholesterol.*

### Diagnostic value of PM20D1 in carotid atherosclerosis

At last, a ROC curve was drawn in order to see the diagnostic value of PM20D1 for CAS, as well as severe CAS or CAS/stroke. It was found PM20D1 could be a potential diagnostic biomarker of CAS with cutoff value of 5.94 ng/ml, area under the ROC curve (AUC) 0.876, 95%CI (0.845~0.906), sensitivity 80.1% and specificity 73.6% (Figure 3). PM20D1 could also be used as a biomarker for severe CAS patients or CAS patients with stroke, with cutoff value of 3.99 ng/ml, AUC 0.917, 95%CI (0.883~0.951), sensitivity 81.6%, specificity 77.2%. Further binary logistic regression which included all factors of age, BMI, CRP, TNF-α, Hcy, TC, TG, LDL-ch, HDL-ch,PM20D1, as well as sex, diabetes, hypertension, history of coronary heart disease, history of stroke and current smoker, showed sex, TNF-α, Hcy and PM20D1 were risk factors for CAS (Table 4).

**Figure 3:** – Receiver operating characteristic (ROC) curves for diagnostic value of PM20D1 of carotid atherosclerosis (CAS), severe CAS or CAS/stroke.

**Table 4:** – Binary logistic regression for risk factors of carotid atherosclerosis

Variables	Wald	Odds ratio	95%CI	p
Age	0.011	1.003	0.935~1.078	0.914
Sex	4.210	0.208	1.073~21.401	0.040
BMI	0.524	1.057	0.909~1.231	0.469
Diabetes	<0.001	<0.001	0.000	0.995
Hypertension	<0.001	<0.001	0.000	0.993
History of coronary heart disease	<0.001	<0.001	0.000	0.995
History of stroke	<0.001	0.001	0.000	0.999
Current smoker	<0.001	0.844	0.232~3.075	0.798
CRP	0.004	1.008	0.777~1.310	0.947
TNF-α	11.820	0.664	0.526~0.839	0.001
Hcy	22.852	0.155	0.073~0.334	<0.001
TC	0.462	0.697	0.248~1.967	0.496
TG	1.403	0.411	0.095~1.787	0.236
LDL-ch	1.559	0.340	0.063~1.847	0.212
HDL-ch	1.216	0.008	1.797E-6~40.532	0.270
PM20D1	18.152	8.485	3.173~22.693	<0.001

*BMI: body mass index; CRP: C-reactive protein; TNF-α: tumor necrosis factor; Hcy: homocysteine; TC: total cholesterol; TG: triglyceride; LDL-ch: low-density leptin cholesterol; HDL-ch: high-density leptin cholesterol.*

## Discussion

Early diagnosis and prediction of CAS can greatly improve the treatment efficacy and prognosis. Thus, biomarkers for diagnosis and prognosis of CAS are of great significance. In the present study, we demonstrated that PM20D1 was down-regulated in CAS patients and was associated with the severity and plaque condition of CAS patients. Besides, PM20D1 was negatively correlated with CRP, TNF-α, Hcy, TC and LDL-ch in CAS patients.

Lipid metabolism and obesity are both associated with development of CAS. It was found that levels of TG, TC and LDL-ch were significantly higher in patients with carotid artery plaque.^16^ Another research found that treatment of atorvastatin or ezetimibe obviously decreased the levels of TC, TG and LDL-ch in CAS patients.^17^ In a recent study, Pan *et al. *showed that, in low-income rural residents in China, LDL-ch and TC were risk factors for early-stage atherosclerosis and carotid plaque risk increased by 24% and 62% for each 1-mmol/L increase in TC and LDL-ch.^18^ In our research, we also found that TG, TC and LDL-ch were highly expressed in CAS patients, especially in severe CAS cases, which was consistent with the aforementioned researches. Besides, we also observed that inflammatory factors such as CRP, TNF-α and expression of Hcy were elevated in CAS patients, which was also demonstrated in several researches.^19-22^

PM20D1 is a factor associated with both lipid metabolism and obesity. It was found that mice with increased circulating PM20D1 showed increased breathing and increased serum N-acyl amino acids, which improved glucose homeostasis and increased energy consumption, and thus might regulate obesity.^13^ Benson et al. demonstrated that decreased PM20D1 was associated with the genetic relationship of obesity and neurodegenerative diseases in humans.^23^ Li et al. found that inhibition of miR-324-5p increased the oxygen consumption of primary white and brown adipose tissue cells, increased fat consumption, and thus reduced the weight of mice by enhancing the levels of PM20D1.^24^ Long et al. observed that, in PM20D1 deficiency mice, knockdown of PM20D1 decreased the N-acetil aspartato hydrolase/synthetase activity in serum and tissues, as well as a variety of metabolic and pain phenotypes, including insulin resistance, cold temperature changes and antinociceptive behavior, which are associated with obesity, diabetes and other diseases.^12^ Thus, we can speculate that higher PM20D1 levels might be beneficial in the improvement of obesity and the reduction of lipid. Although there is no study demonstrating the role of PM20D1 in CAS, the down-regulation of PM20D1 in CAS patients in our study might be partly due to the dysfunction of lipid metabolism and obesity of CAS patients. Since CAS patients showed lower PM20D1 levels than healthy control who had the same BMI distribution, and CAS patients with different BMI showed no significant difference of PM20D1, this result indicated that BMI might not be associated with the level of PM20D1 in CAS patients. The negative correlation between PM20D1 and Hcy, TC and LDL-ch might be one of the reasons for the abnormal expression of PM20D1 in CAS patients. However, to substantiate all these speculations, more studies are needed in order to obtain further evidence.

The present study has some limitations. First, this is an observational study from a single center, with only 231 cases. Secondly, molecular mechanism for how PM20D1 influences CAS development is still unclear.

## Conclusion

In conclusion, this observational study demonstrated that decreased PM20D1 was associated with the severity, plaque condition, and expression of CRP, TNF-α, Hcy, TG, TC and LDL-ch in CAS patients. This study might provide novel research target for PM20D1 in CAS.
